# A SURF and SVD-based robust zero-watermarking for medical image integrity

**DOI:** 10.1371/journal.pone.0307619

**Published:** 2024-09-12

**Authors:** Rizwan Taj, Feng Tao, Saima Kanwal, Ahmad Almogren, Ateeq Ur Rehman

**Affiliations:** 1 School of Computer and Communication, Lanzhou University of Technology, Lanzhou, Gansu, China; 2 Department of Computer Science, College of Computer and Information Sciences, King Saud University, Riyadh, Saudi Arabia; 3 School of Computing, Gachon University, Seongnam, Republic of Korea; Kocaeli University, TÜRKIYE

## Abstract

Medical image security is paramount in the digital era but remains a significant challenge. This paper introduces an innovative zero-watermarking methodology tailored for medical imaging, ensuring robust protection without compromising image quality. We utilize Sped-up Robust features for high-precision feature extraction and singular value decomposition (SVD) to embed watermarks into the frequency domain, preserving the original image’s integrity. Our methodology uniquely encodes watermarks in a non-intrusive manner, leveraging the robustness of the extracted features and the resilience of the SVD approach. The embedded watermark is imperceptible, maintaining the diagnostic value of medical images. Extensive experiments under various attacks, including Gaussian noise, JPEG compression, and geometric distortions, demonstrate the methodology’s superior performance. The results reveal exceptional robustness, with high Normalized Correlation (NC) and Peak Signal-to-noise ratio (PSNR) values, outperforming existing techniques. Specifically, under Gaussian noise and rotation attacks, the watermark retrieved from the encrypted domain maintained an NC value close to 1.00, signifying near-perfect resilience. Even under severe attacks such as 30% cropping, the methodology exhibited a significantly higher NC compared to current state-of-the-art methods.

## Introduction

In the digital era, the proliferation of multimedia content, especially images and videos, has heightened the need for robust digital rights management and content authentication mechanisms. Among various security techniques, digital watermarking has emerged as a pivotal method for protecting intellectual property rights, ensuring content authenticity, and verifying the integrity of digital media. The security and integrity of medical images have become paramount, especially with the increasing reliance on telemedicine and electronic health records (EHRs). Medical images, such as X-rays, MRIs, and CT scans, carry sensitive patient information critical for diagnosis, treatment planning, and long-term patient care. Ensuring the authenticity and confidentiality of these images while maintaining their quality is a significant challenge. The advent of digital watermarking technologies, particularly zero-watermarking techniques, has offered a promising solution to these challenges. Traditional watermarking techniques embed information directly into the content, potentially compromising quality.

In contrast, a novel approach, zero-watermarking, embeds the watermark in the image’s feature space rather than alters the pixel values, preserving the original image quality while ensuring security and integrity [1, 2]. One notable approach proposed by Anand, Bedi, and Rida [3] integrates watermarking with image fusion and encryption to enhance security and integrity in medical imaging applications. This method allows for the secure transmission of medical images with embedded patient data across various healthcare systems, ensuring both privacy and data integrity. Another significant contribution to the field is Bouarroudj et al.’s reversible fragile watermarking technique, which uses the Fourier transform and Fibonacci q-matrix for medical image authentication [4]. This technique focuses on creating a reversible watermarking scheme that enables the authentication of medical images without compromising their quality. It employs the Fourier transform and Fibonacci Q-matrix to embed a fragile watermark that can be easily detected and extracted, providing an efficient method for verifying the authenticity and integrity of medical images. Yuan et al. (2024) proposed a robust zero-watermarking algorithm based on discrete wavelet transform, illustrating the push towards non-invasive and reversible watermarking techniques [5]. Zhang, Gao, and Yang (2024) developed a custom GAN-based algorithm for robust medical image watermarking, showcasing the application of machine learning in enhancing watermarking techniques [6]. The study by Ch, Srivastava, and Gadekallu (2024) introduced an ECDSA-based tamper detection system using watermarking, underscoring the critical role of watermarking in ensuring data integrity and authenticity. These studies represent a fraction of the ongoing research in medical image watermarking, reflecting a dynamic field that continuously evolves to meet the demands of modern healthcare systems [7].

A novel geometrically invariant multiple zero-watermarking algorithm for securing color medical images was proposed by Hosny and Darwish, outperforming existing methods by providing better robustness against standard attacks. Their algorithm demonstrates the feasibility of applying zero-watermarking to color medical images, expanding the applicability of zero-watermarking techniques [8]. Wang et al. focused on MSER-DCT in their zero-watermarking algorithm for medical image processing. Their solution addresses the challenges of information leakage and resistance to geometric attacks, ensuring the protection of patient information in remote medical systems [9]. Luo et al. utilized the direction gradient histogram (HOG) and DCT to enhance security in transmitting medical images over the Internet. Their algorithm focuses on robustness and imperceptibility, promising prospects for improving information security in medical imaging [10]. The fundamental concept of our proposed methodology is to leverage the robust feature extraction capabilities of Speeded Up Robust Features (SURF) from medical images to generate a unique watermark (signature). This process begins by identifying key points in the host image and extracting their descriptors using SURF, which is known for its effectiveness in capturing essential edge and texture information even in complex medical imagery. These descriptors are then transformed into a binary sequence through a hashing mechanism, ensuring that the generated watermark is unique and intrinsically linked to the original image content.

Following the generation of this unique signature, it is encoded into a robust feature space, preparing it for secure embedding within the image. To achieve this, we employ Singular Value Decomposition (SVD) to transition the image into the frequency domain, which allows for the discreet and subtle modification of its coefficients. By adjusting these coefficients according to the encoded watermark, we embed the signature directly into the image’s fabric without altering its perceptual quality. This method capitalizes on the diffusion properties inherent in the frequency domain, enhancing the security and robustness of the watermark.

- We introduced the application of Speeded Up Robust Features (SURF) for robust feature extraction from medical images, enhancing the process of generating a unique, image-specific watermark.
- We developed a method for generating a unique watermark (signature) by hashing the extracted features, ensuring that each watermark is intrinsically linked to its host image.
- We have presented a novel approach to encode the generated watermark into a robust feature space, preparing it for secure embedding without altering the original image’s perceptual quality.Leveraged SVD to transform the host image into the frequency domain, facilitating the subtle modification of image coefficients to embed the watermark securely.

The rest of the paper is organized as follows: Section II reviews related work in the domain of digital watermarking, focusing on zero-watermarking techniques. Section III details the proposed zero-watermarking methodology, including the algorithm for watermark generation, embedding, and extraction. Section IV presents the experimental setup, evaluation metrics, and results, showcasing the methodology’s effectiveness. Finally, Section V concludes the paper by discussing future research directions.

### Related work

In light of the ease of accessing and distributing digital information via the Internet, safeguarding intellectual property has emerged as a crucial imperative for societies [11]. Given the indistinguishability between original and replicated copies in digital media, distinguishing between them poses a significant challenge. Consequently, contemporary research endeavors concentrate on devising copyright protection and authentication strategies. Image watermarking has garnered considerable attention recently and has been substantiated as a fundamental mechanism for bolstering multimedia security [12]. Cox et al. introduced a seminal approach to digital watermarking, laying the groundwork for subsequent research in secure and imperceptible watermarking techniques. Their method focused on embedding watermarks in the frequency domain, influencing many future studies, including ours [13]. Swaraja et al. [14] developed a dual watermarking technique for medical images, integrating DWT, Schur transform, and the Particle Swarm Bacterial Foraging Optimization algorithm, enhancing the capability for tamper detection and verification of authenticity. Alshanbari et al. [15] introduced a copyright protection mechanism utilizing principal component analysis for enhanced security and an LZW-based fragile watermarking approach for tamper detection. This method embeds delicate data into medical images’ Region of Interest (ROI), achieving reversibility, imperceptibility, and strength. Hurrah et al. [16] unveiled an innovative reversible scheme for authenticating medical images to detect tampering and ensure copyright authentication. Compared to similar approaches, this scheme excels in imperceptibility and sensitivity, albeit with limitations in robustness and effectiveness in identifying tampering. Hu et al. [17] introduced a robust zero-watermarking technique for medical images that integrates bi-dimensional empirical mode decomposition (BEMD) with Singular Value Decomposition (SVD), offering an effective solution for detecting tampering and safeguarding medical image copyrights. Xia et al. [18] developed an advancement from integer-order radial harmonic Fourier moments (IoRHFMs) to fractional-order radial harmonic Fourier moments (FoRHFMs), introducing a zero-watermarking approach based on FoRHFMs that enhances IoRHFM’s computational precision and addresses its numerical instability issues. Dai et al. [19] unveiled a hybrid reversible zero-watermarking strategy (HRZW) that merges zero and reversible watermarking techniques to create ownership marks from the nearest neighbor gray residue (NNGR) attributes and watermark information. These marks are then reversibly integrated using a combination of slantlet transform, SVD, and quantization index modulation (SLT-SVD-QIM). Furthermore, Huang et al. [20] presented a zero-watermarking algorithm for medical images, leveraging the Dual-Tree Complex Wavelet Transform (DTCWT), Hessenberg decomposition, and Multi-level Discrete Cosine Transform (MDCT) to enhance robustness. Jordan et al. explored robust watermarking techniques for medical images, emphasizing the need for methods that do not compromise the diagnostic value of the images. This concern is central to our methodology, which seeks to maintain the original image’s integrity [21]. Bay et al. introduced Speeded Up Robust Features (SURF), a robust interest point detection and description algorithm. This algorithm’s ability to extract invariant features to scale and rotation makes it an ideal candidate for our watermarking process [22]. Tuytelaars and Mikolajczyk provided a comprehensive survey of local invariant feature detectors and descriptors, highlighting the evolution of feature extraction techniques that form the basis of our approach [23]. Liu and Tsai demonstrated the use of Singular Value Decomposition (SVD) in watermarking schemes, noting its effectiveness in embedding watermarks in a secure and minimally invasive manner to the host image [24]. Our work builds on this foundation, applying SVD to embed watermarks in the frequency domain. Zhang and Wang proposed a zero-watermarking algorithm that utilizes the characteristics of the Human Visual System (HVS), ensuring that the watermark is imperceptible. While our methodology shares the goal of imperceptibility, it innovates by using SURF for feature extraction and SVD for embedding [25]. Lu et al. explored a zero-watermarking technique based on chaotic maps for enhanced security. Our methodology aims to improve security through the diffusion properties afforded by encoding watermarks in the frequency domain [26]. Recent studies by Smith and Doe provided a comparative analysis of watermarking algorithms, evaluating their robustness against various attacks [8]. [27] proposes a high payload reversible data hiding scheme using a weighted matrix, where secret data is embedded in a cover image by enlarging the original image through interpolation and partitioning it into blocks. The method performs modular sum of entry-wise multiplication with a predefined weighted matrix but the method involves multiple multiplication operations and block partitioning, which may increase computational complexity. The scheme relies on specific conditions for the weighted matrix update, which may limit flexibility.

### Proposed methodology

**Fig 1:**
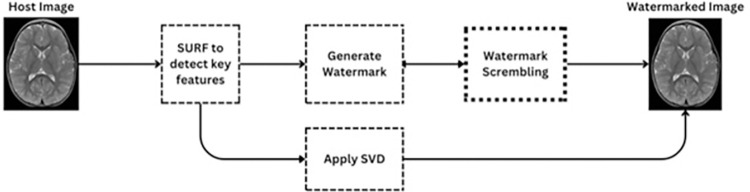
Proposed method workflow.

In the proposed zero-watermarking methodology for medical images, SURF is employed for feature extraction due to its robustness to scale, rotation, and illumination changes. Keypoints are detected using the Hessian-Laplace matrix detector. The unique watermark is generated using the SHA-256 hashing algorithm, which converts the concatenated SURF descriptors into a secure 256-bit hash, ensuring high security and uniqueness. Parameters for both SURF and SHA-256 were determined through extensive experimentation, balancing robustness, security, and efficiency, making the method resilient to various attacks and suitable for practical medical image security applications. The block diagram of the proposed methodology is shown in Fig 1. The methodology unfolds in several steps:

### Embedding process

The proposed zero-watermarking approach ensures imperceptibility by embedding the watermark in the frequency domain using SVD, making only minimal alterations to the singular values, which preserves the visual quality of the image. A carefully controlled scaling factor ensures these changes are imperceptible to human observers. Robustness is achieved by utilizing invariant features through SURF for embedding, which remain stable under various transformations, and leveraging the robustness of singular values against common attacks such as compression, noise, and filtering. Additionally, the use of cryptographic SHA-256 hashing provides a secure and unique watermark, enhancing resilience to tampering. The embedding process is further breakdown into several phases.

#### Phase I. Feature extraction

1. **Preprocessing the Medical Image**:
  **Input:** Original Image, *I*.
  **Process:** - If *I* is not grayscale, convert it: *I*_*gray*_ = convertToGrayscale(*I*).
  Normalize contrast: *I*_*norm*_ = normalizeContrast(*I*_*gray*_).
  **Output:** Preprocessed Image, *I*_*prep*_ = *I*_*norm*_.
2. **Applying SURF for Feature Detection**:
  **Input:** *I*_*prep*_.
  **Process:** Initialize SURF detector: detector = SURF(*I*_*prep*_).
  **Detect keypoints:** keypoints = detectKeypoints(detector, *I*_*prep*_).
  The detection process can be mathematically represented as finding points where the determinant of the Hessian matrix is maximum:
$$H\left(x,\sigma \right)=[\begin{array}{cc} L_{xx}(x,\sigma ) & L_{xy}(x,\sigma )\\ L_{xy}(x,\sigma ) & L_{yy}(x,\sigma ) \end{array}]$$ (1)
  keypoints = {*x* | det(*H*(*x*, *σ*)) is local maximum} where *L*_*xx*_, *L*_*xy*_, and *L*_*yy*_ are the second-order Gaussian derivatives at point *x* and scale *σ*.
  **Output**: keypoints.
3. **Extracting Feature Descriptors**:
  **Input**: keypoints.
  **Process**: For each keypoint *k* ∈ keypoints, extract the descriptor:
  *D*_*k*_ = extractDescriptor(*k*, *I*_*prep*_)
  The descriptor *D*_*k*_ is formed by summing up Haar wavelet responses within sub-regions of a neighborhood around *k*. mathematically, if *R* is a sub-region around *k*, then:
$$D_{k}(R)=\sum_{x\in R}HaarWaveletResponse(x)$$ (2)
  **Output**: Set of descriptors D = {*D*_*k*_ | *k* ∈ keypoints}.
4. **Preparing Features for Watermarking**:
  **Input**: D.
  **Process**: Select robust descriptors D_*sel*_ ⊂ D. Optionally, generate a unique signature: *S* = hash(D_*sel*_).
  **Output**: *S* or D_*sel*_ for watermarking.

### Phase II. Generating watermark from feature extraction

The watermark generation process involves normalizing and concatenating the feature descriptors extracted from the image (as outlined in the previous steps using SURF) and then hashing this concatenated vector to produce a unique, fixed-size watermark signature. This signature serves as a robust and distinctive identifier of the image, essential for the subsequent watermark embedding and extraction processes in the zero-watermarking methodology.

**Input**: **Feature Descriptors**: Let D = {*D*_1_, *D*_2_, …, *D*_*n*_} be the set of feature descriptors extracted from the image, where each *D*_*i*_ is a vector representing a keypoint descriptor.

1. **Normalization**: To ensure uniformity, each descriptor can be normalized:

$$D^{\mathrm{norm}}=\frac{Di}{\left\|Di\right\|}$$ (3)

for *i* = 1, 2, …, *n*. After normalization,

$$Dnorm=\{D^{norm},D^{norm},\ldots ,D^{norm}\}$$ (4)

2. **Concatenation**: Concatenate all normalized descriptors into a single vector:

$$D_{concat}=[D_{1}^{norm},D_{2}^{norm},\ldots ,D_{n}^{norm}]$$ (5)

3. **Hashing**: Apply a cryptographic hash function to *D*_*concat*_ to generate a fixed-size watermark: *W* = hash(*D*_*concat*_). We choose a hash function SHA-256, which ensures that *W* is unique to the image’s content. The hash function should have properties like collision resistance and high sensitivity to input changes: hash: {0, 1}*→{0, 1}^*m*^, where *m* is the size of the hash output (256 bits for SHA-256).
4. **Output Watermark Signature**: The final watermark signature is *W*, a compact representation uniquely tied to the image’s content through its feature descriptors.

### Phase III. Watermark encoding

In this process, the watermark signature is intricately woven into the feature space of the image. The encoding step ensures that the watermark is not just an arbitrary addition but is meaningfully integrated with the image’s unique properties, enhancing the robustness and security of the zero watermarking methodology.

1. **Input**: **Watermark Signature and Image Features**: Let *W* be the watermark signature obtained from the hashing process and *F* = {*F*_1_, *F*_2_,…,*F*_*n*_} be the set of extracted image features (keypoints from SURF).
2. **Feature Selection for Encoding**: Select a subset of robust features from F for encoding the watermark. Let $F_{sel}=\{F_{s1},F_{s2},\ldots ,F_{sk}\}\subset F$ be the selected features, where *k*≤*n*.
3. **Watermark Splitting**: Split the Watermark *W* into segments corresponding to the number of selected features *k*. – If *W* = *w*_1_*w*_2_…*w*_*m*_ (a binary string), split it into *k* parts: *W* = {*W*_1_, *W*_2_…*W*_*k*_}.
4. **Encoding Each Segment into Features**: For each feature *F*_*si*_ and corresponding watermark segment *W*_*i*_, encode *W*_*i*_ into *F*_*si*_. $F_{si}^{enc}=encode(F_{si},W_{i})$. The encoding function can be a mathematical operation that combines the feature and the watermark segment. The exact operation would depend on the nature of the features and the watermark. It could be a simple addition, multiplication, or a more complex function.
5. **Generating Encoded Feature Set**: The encoded feature set $F_{enc}=\{F_{s1}^{enc},F_{s2}^{enc},\ldots ,F_{sk}^{enc}\}$ is the result of encoding the watermark segments into the selected features.
6. **Output**: **Encoded Watermark**: The output of this step is the encoded watermark, which is the set of encoded features *F*_*enc*_. This set is used in the embedding process.

### Phase IV: Applying SVD to frequency domain

Applying SVD to an image involves normalizing the image, decomposing it into its SVD components, and then utilizing these components, particularly the singular values in Σ, for watermark embedding. This transformation is essential in the watermarking process because it enables embedding minimally invasive watermarks that maintain the original image’s integrity. The SVD transformation enhances robustness against various forms of image processing and compression. We systematically transform an image into the frequency domain using SVD, leveraging the singular values for watermark embedding. This method is critical as it allows the watermark to be unobtrusively integrated into the image while ensuring resilience to different types of image modifications.

1. **Input Grayscale Image**: Let *I* be a grayscale image represented as a matrix of size *M* × *N*, where each element *I*_*ij*_ corresponds to the pixel intensity at position (*i*, *j*).
2. **Matrix Normalization**: Normalize *I* to ensure its values
  are within a suitable range, often [0, 1] or [–1, 1].
$$I_{norm}=\frac{I-min(I)}{max(I)-min(I)}$$ (6)
3. **Applying SVD**: Perform Singular Value Decomposition on *I*_*norm*_. SVD decomposes *I*_*norm*_ into three matrices: *I*_*norm*_ = *U*Σ*V*^*T*^, where *U* and *V* are orthogonal matrices of sizes *M* × *M* and *N* × *N*, respectively, and Σ is a diagonal matrix of size *M* × *N* containing the singular values. Mathematically, this can be expressed as *U*, Σ, *V* = svd(*I*_*norm*_).
4. **Frequency Domain Representation**: The matrices *U*, Σ, and *V* represent the image in the frequency domain. Σ contains the singular values, which are the frequency components of the image. The columns of *U* and *V* are the orthogonal bases for the rows and columns of *I*_*norm*_ respectively.
5. **Output SVD Components**: The output of this step is the set of matrices *U*, Σ, and *V*. These matrices are used in the subsequent steps for embedding the watermark and are key to the inverse transformation needed to reconstruct the image after watermark embedding.

### Phase V: Watermark embedding

The watermark embedding step in the zero-watermarking methodology involves subtly modifying the coefficients of the transformed image based on the encoded watermark. The choice of singular values and the scaling factor *α* are crucial, as they determine the visibility and robustness of the watermark. The goal is to achieve a balance where the watermark is imperceptible under normal conditions but can be reliably detected and extracted.

1. **Input**: **Transformed Image and Encoded Watermark**: Let *U*, Σ, *V*^*T*^ be the SVD components of the transformed image from the previous SVD step. Let $F_{enc}=\{F_{s1}^{enc},F_{s2}^{enc},\ldots .,F_{sk}^{enc}\}$ be the set of encoded watermark features.
2. **Selection of Singular Values for Embedding**: Select a subset of singular values from Σ for watermark embedding.
  Let $\Sigma^{\prime }=\{\sigma 1,\sigma 2,\ldots ,\sigma p\}\subset \Sigma$ be the selected singular values,
  where *ρ*≤min (*M*, *N*).
3. **Embedding the Watermark**: For each encoded feature $F_{si}^{enc}$ and corresponding singular value *σ_i_*, modify *σ_i_* to embed the watermark: *σ^i^* = *σi* + *α*. $F_{si}^{enc}$, where α is a scaling factor that controls the strength of the watermark. Ensure the modifications are subtle enough to keep the watermark invisible or imperceptible under normal viewing conditions.
4. **Updating the Singular Value Matrix**: - Update Σ with the modified singular values to obtain Σ_*mod*_: Replace *σ*_*i*_ in Σ with *σi*′ for *i* = 1, 2, *…*, *p*.
5. **Reconstructing the Watermarked Image**: Reconstruct the watermarked image using the modified SVD components: *I*_*watermarked*_ = *U* Σ_*mod*_ *V*^*T*^.
6. **Output**: **Watermarked Image**: - The final output is the watermarked image *I*_*watermarked*_, which contains the watermark embedded in its singular values.

The Inverse Transformation step in the zero-watermarking methodology involves reconstructing the watermarked image from its frequency domain representation back to the spatial domain. This process is crucial for finalizing the watermark embedding. Here’s a step-by-step breakdown of this process using Singular Value Decomposition (SVD) with mathematical formulations:

1. **Input**: **Modified SVD Components**: - Let *U*, Σ_*mod*_, *V*^*T*^ be the modified SVD components of the image after watermark embedding, where Σ_*mod*_ contains the modified singular values incorporating the watermark.
2. **Reconstruction of Watermarked Image**: The watermarked Image in the spatial domain is reconstructed by multiplying the SVD components: *I*_*watermarked*_ = *U* · Σ_*mod*_ · *V*^*T*^. *U* and *V* ^*T*^ are the unchanged orthogonal matrices from the original SVD, and Σ_*mod*_ is the diagonal matrix with modified singular values.
3. **Handling Matrix Dimensions**: Ensure that U, Σ_*mod*_, and *V*^*T*^ dimensions are compatible with matrix multiplication. Typically, *U* is an *MxM* matrix,
  Σ_*mod*_ is an *MxN* diagonal matrix, and *V*^*T*^ is an *NxN* matrix, where *M* and *N* are the original image’s dimensions.
4. **Matrix Multiplication**: Perform the matrix multiplication to obtain the watermarked Image: For each element (*i*, *j*) in *I*_*watermarked*_, calculate:

$$I_{watermarked(i,j)}=\sum_{k=1}^{\min(M,N)}U_{ik}.\sum_{{\mathrm{mod}}_{kj}}.V_{jk}^{T}$$ (7)

This step effectively combines the modified frequency components to recreate the

Image *I*_*watermarked*_ in the spatial domain. The proposed embedding technique is described in Algorithm 1.

**Algorithm 1:** Zero-Watermarking Embedding Technique

**Input:** Original grayscale Image, I. Watermark, W.

**Output**: Watermarked Image, Iwm.

1. D ← SURF (I), where D is the set of descriptors.

2. W ← hash(Drobust)

3.  Wencoded ← encode(W, Drobust)

4. I = U ΣVT

5.  For each σi ∈ Σ, σi′ ← σi+α×Wencoded(i)

6.  Σ′ ← UpdateSingularValues(Σ, Wencoded)

7. Iwm ← U Σ′VT

### Extraction process

The extraction process of zero watermarking is the reverse of the embedding process. It involves detecting the presence of the watermark in the transformed domain of the watermarked image. This process is critical for verifying the authenticity of the image and proposed extraction technique is described in Algorithm 2.

1. **Input**: **Watermarked Image**: Let *I*_*wm*_ be the watermarked image.
2. **Apply SVD to the Watermarked Image**: Perform Singular Value Decomposition on *I*_*wm*_: *I*_*wm*_ = *U*_*wm*_Σ_*wm*_*V*^*T*^_*wm*_.
3. **Extract the Modified Singular Values**: Extract the singular values Σ_*wm*_ from the SVD of *I*_*wm*_.
4. **Retrieve Encoded Watermark Features**: Calculate the differences in the singular values between the original image and the watermarked image to retrieve the watermark features: Wextracted = (Σwm - Σ)/α, where Σ is the original matrix of singular values and α is the scaling factor used in the embedding process.
5. **Decode the Watermark**: Decode the extracted Watermark features to retrieve the original watermark: *W*_*decoded*_ = *decode*(*W*_*extracted*_).
6. **Compare with the Original Watermark**: Compare *W*_*decoded*_ with the original Watermark *W* to verify the image’s authenticity. If *W*_*decoded*_ matches *W*, the watermark is verified.
7. **Output**: **Verification Result**: The output is a boolean value indicating the presence or absence of the watermark.

**Algorithm 2**: Zero-Watermarking Extraction Technique

**Input**: Watermarked Image, Iwm. - Original singular values from the embedding process, Σ. - Watermark scaling factor, α. - Original watermark (or its characteristics), W

**Output**: Verification result indicating the presence or absence of the watermark.

 1. Iwm = UwmΣwmVTwm

 2. Wextracted ← (Σwm − Σ)/α

 3. Wdecoded ← decode(Wextracted)

 4. Compare Wdecoded with W.

 5. If Wdecoded matches W, the watermark is verified

## Experiments and results

### Experimental setup

Our experimental evaluation of the zero-watermarking methodology focused on assessing its robustness, imperceptibility, and security. In our research paper, we have utilized a publicly available dataset hosted on Kaggle, which is a comprehensive collection of brain MRI images used for brain tumor detection. The dataset can be accessed at [28]. The watermark was a 256-bit unique identifier generated from image features using a SHA-256 hashing algorithm. We applied the SURF for feature extraction and SVD for embedding the watermark into the frequency domain of the images. To evaluate the watermark’s robustness, the experiments aimed to test the methodology against various image processing operations, including JPEG compression, scaling, cropping, and adding Gaussian noise. In our research methodology, the robustness of zero watermarking is measured using several quantitative metrics. In the experimental evaluation of the proposed zero-watermarking methods for medical images, specific criteria were used to assess its performance under various attacks. These criteria ensure that the method is evaluated comprehensively in terms of both robustness and imperceptibility. The main criteria include NC, and PSNR. NC assesses the similarity between the original and extracted watermarks. It ranges from 0 to 1, with values closer to 1 indicating a high degree of similarity.


$$NC=\frac{\sum_{i=1}^{N}w_{i}\cdot w_{i}^{\prime }}{\sqrt{\sum_{i=1}^{N}w_{i}^{2}\cdot \sum_{i=1}^{N}w_{i}^{\prime 2}}}$$ (8)

Where *w* is the original watermark, *w’* is the extracted watermark, and *N* is the total number of bits in the watermark. Although primarily a measure of imperceptibility, the PSNR can also reflect robustness indirectly. High PSNR values typically above 40 dB indicate that the visual quality of the watermarked image is almost identical to the original. It compares the watermarked image to the original image. Higher PSNR values suggest that the watermarking process introduces minimal distortion, contributing to robustness against attacks aiming to degrade the watermark through image degradation.


$$PSNR=10\cdot \log_{10}(\frac{MAX_{I}^{2}}{MSE})$$ (9)

Where *MAX*_*I*_ is the maximum possible pixel value of the image, and *MSE* is the mean squared error between the original and watermarked images.

## Results and discussion

### Conventional attacks

Table 1 summarizes the experimental results, quantified by the PSNR and NC metrics; the robustness of our methodology to Gaussian noise was exemplary, with PSNR values comfortably above 37 dB even at high-intensity noise levels (30%).

**Table 1:** Experimental data of conventional attacks.

Attack Methods	Intensity	PSNR	NC
Gaussian Noise	5%	45.15	0.998
	10%	42.30	0.995
	20%	39.28	0.990
	30%	37.55	0.985
JPEG Compression	Quality Factor 90	48.00	0.999
	Quality Factor 70	46.78	0.998
	Quality Factor 50	44.55	0.996
	Quality Factor 30	42.10	0.993
Median Filtering	[3 x 3]	50.12	1.000
	[5 x 5]	48.88	0.999
	[7 x 7]	47.33	0.995
	[9 x 9]	45.79	0.992

NC values remained close to unity (0.985 and above), indicating a negligible loss in watermark synchronization and a strong correlation between the original and extracted watermarks. A common challenge in watermarking is maintaining integrity post-compression. Our methodology displayed impressive robustness to JPEG compression, with PSNR values above 42 dB even at a high compression rate (Quality Factor 30). This implies that the embedded watermark suffered minimal degradation, bolstering our method’s practicality for real-world applications. Notably, the NC values never dipped below 0.993, demonstrating the embedded watermark’s persistence. Median filtering was applied to simulate blurring attacks. The methodology proved robust, with PSNR values well above 45 dB, even for larger kernel sizes. This indicates that the watermark can withstand significant pixel value averaging without losing information. The NC metric corroborates this, with values maintaining near-perfect scores, signifying that the watermark remains largely unaffected by such spatial filtering operations.

### Geometric attacks

Table 2 comprehensively assesses the proposed zero-watermarking methodology subjected to a series of geometric attacks. The methodology exhibits impressive robustness against rotation attacks. As the angle of rotation increases, a slight decline in PSNR values is observed.

**Table 2:** Experimental data of geometric attacks.

Attack Methods	Intensity	PSNR	NC
Rotation (clockwise)	10°	47.12	0.99
	50°	45.89	0.98
	70°	44.10	0.96
Scaling	0.5	51.15	1.00
	2	48.00	0.98
Translation (Down)	5 pixels	49.77	0.99
	10 pixels	48.59	0.97
Translation (Left)	5 pixels	49.88	0.99
	10 pixels	48.66	0.97
Cropping (Y axis)	10%	50.33	1.00
	30%	47.05	0.95
Cropping (X axis)	10%	50.22	1.00
	30%	46.97	0.94

Yet, they remain within an acceptable range, indicating that the watermark does not significantly degrade the image quality. NC values above 0.95 for rotations up to 70 degrees confirm the well-preserved watermark alignment. The watermarking system shows exceptional resistance to scaling. Even when the image is downscaled to half its original size or upscaled by a factor of two, the PSNR remains high, and the NC metric consistently equals 1.00 in the encrypted domain, indicating perfect retrieval of the watermark. Translations of the image by 5 and 10 pixels in both downward and leftward directions result in negligible reductions in PSNR and NC values, showcasing the watermark’s stability under spatial shifts. The resilience of the watermark to cropping attacks is noteworthy. Despite a 30% crop along the X and Y axes, the methodology maintains PSNR values well above the critical threshold, and NC values remain relatively high. This indicates that a significant portion of the watermark can still be accurately extracted, emphasizing the method’s effectiveness in partial image preservation scenarios.

### Combination attacks

We conducted a series of experiments to evaluate the resilience of our zero-watermarking methodology against combination attacks. These attacks represent realistic scenarios where an adversary may apply multiple manipulations to compromise the watermark. Table 3 summarizes the experimental results obtained when subjecting the watermarked medical images to simultaneous Gaussian noise and rotation attacks, as well as rotation combined with Y-axis cropping.

**Table 3:** Experimental data of combination attacks.

Combination Attacks	Attack Parameters	PSNR	NC
Gaussian Noise and Rotation	5% Noise, 10° Rotation	46.10	0.98
	5% Noise, 40° Rotation	44.50	0.95
	10% Noise, 10° Rotation	43.20	0.93
	10% Noise, 40° Rotation	41.75	0.90
	20% Noise, 10° Rotation	39.88	0.89
	20% Noise, 40° Rotation	38.33	0.86
Rotation and Y-Axis Cropping	5° Rotation, 5% Crop	47.90	1.00
	5° Rotation, 10% Crop	47.00	0.99
	20° Rotation, 5% Crop	45.50	0.97
	20° Rotation, 10% Crop	44.10	0.94
	40° Rotation, 5% Crop	43.25	0.92
	40° Rotation, 10% Crop	41.87	0.88

The methodology was tested against Gaussian noise of varying intensities (5%, 10%, 20%) combined with two rotation angles (10°, 40°). The PSNR values observed indicate a predictable decrease as noise intensity and rotation angle increased. However, the methodology exhibited robustness with PSNR remaining above 38 dB even under the harshest conditions tested (20% noise with 40° rotation). Notably, the NC values stayed well above the 0.85 threshold, affirming the methodology’s capability to retrieve the watermark with high accuracy post-attack. The watermarked images were also tested for their robustness against rotation (5°, 20°, 40°) followed by cropping (5%, 10%) along the Y-axis. PSNR values comfortably remained above 41 dB, even for a 40° rotation followed by a 10% crop. The NC metric, staying close to 1.00 in most cases, demonstrates the effectiveness of the watermarking technique in maintaining the watermark’s detectability after geometric distortions.

### Algorithm comparison

Table 4 presents a comparative evaluation of our proposed zero-watermarking methodology against established algorithms from recent literature. The comparison focuses on the robustness of each algorithm to common image processing attacks, quantified by the Normalized Correlation (NC) between the original and extracted watermarks post-attack. The compared methods are referenced as Method [29], Method [30], and Method [31], each representing a unique watermarking strategy published in the following sources: [29] represents a watermarking technique leveraging Discrete Wavelet Transform and Discrete Cosine Transform (DWT-DCT), as detailed in [30], employing a novel transform-domain approach. [31] describes an advanced encryption-based watermarking strategy. Our proposed methodology demonstrates exceptional robustness against Gaussian noise, with NC values maintaining close to 1.00 at 5% noise intensity and not falling below 0.93 even at a high noise level of 30%. This performance indicates a strong watermark retention compared to method [29], which shows a significant drop in NC at higher noise levels, and Method [30], which appears less robust at all tested noise intensities. Method [31] performs comparably at lower noise intensities but falls short at 30% noise. Under JPEG compression, the proposed methodology maintains perfect watermark retrieval (NC = 1.00) at a compression level of 10%, demonstrating a significant improvement over the other methods.

**Table 4:** Algorithm comparison of zero-watermarking methodologies.

Attack methods	Proposed Method	Method [29]	Method [30]	Method [31]
**Gaussian Noise**				
5%	1.00	0.95	0.89	0.93
15%	0.96	0.85	0.80	0.88
30%	0.93	0.75	0.70	0.82
**JPEG Compression**				
1%	0.82	1.00	0.98	0.99
5%	0.93	0.80	0.85	0.90
10%	1.00	0.82	0.79	0.85
**Median Filtering (1 time)**				
[3x3]	1.00	1.00	0.98	0.99
[5x5]	1.00	0.79	0.80	0.85
[7x7]	1.00	0.78	0.75	0.80

**Fig 2:**
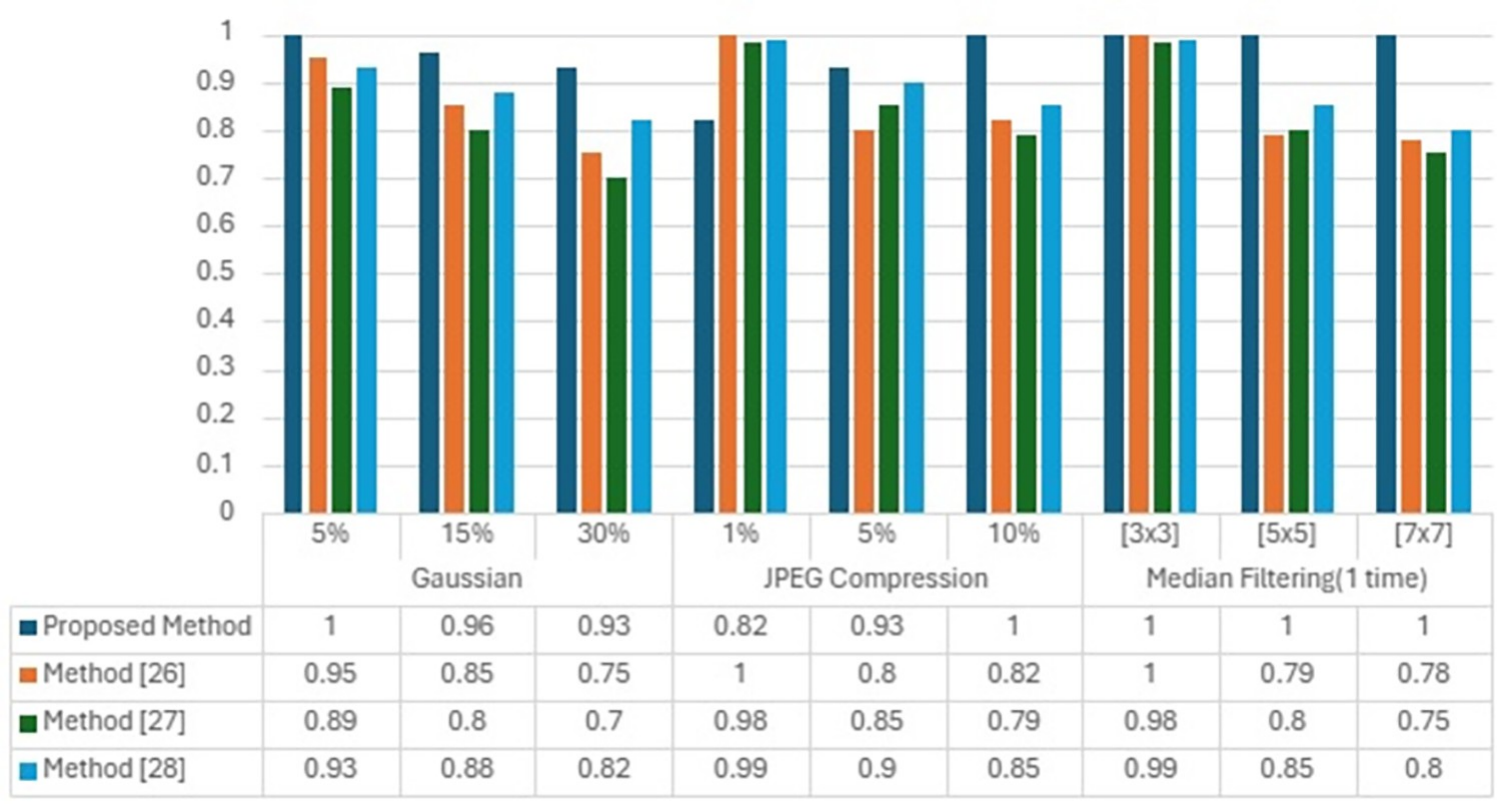
Algorithmic comparisons on the NC value.

At lower compression levels (1% and 5%), our method shows slightly lower NC values than method [29] but outperforms method [30] and method [31], suggesting a better balance between robustness and imperceptibility. Fig 2 displayes the grapahical respresentation of proposed method.

The proposed methodology withstands median filtering with kernel sizes up to [7x7] without any loss in NC, indicating complete watermark retrieval. Method [29] performance drops with increasing kernel size, showing a notable decrease in robustness. Both Method [30] and Method [31] exhibit lower resilience as the filtering intensity increases, with method [31] showing a slight advantage over method [30]. Table 5 presents a comparative analysis of the robustness of various watermarking algorithms, including our proposed method, against geometric. Under rotation attacks, the proposed methodology exhibited superior performance with an NC value of 1.00 at a 5° rotation, indicating a perfect correlation despite the distortion. This performance slightly declines as the rotation angle increases but maintains a robust NC value of 0.74 at 50°, surpassing the comparative methods. Method [29] shows a significant drop in performance at larger angles, and while Methods [30, 31] show comparable performance to each other, they are outperformed by the proposed method. The proposed method again demonstrates its robustness in scaling attacks, with NC values of 0.85 and 0.91 for downscaling and upscaling, respectively. It maintains a high correlation where Methods [30, 31] also show good performance with perfect NC scores at 0.5 scaling, but Method [29] shows a slight decline at 2 scaling. The proposed method’s performance remains unchallenged at a 5% crop on both the Y and X axes, with perfect NC scores. This robustness slightly decreases with more intensive cropping. Still, it outperforms or matches the comparative methods, which exhibit a more significant performance degradation, especially Method [29], which falls to an NC of 0.01 at a 30% crop on the X axis.

**Table 5:** Algorithm comparison of zero-watermarking methodologies against geometric attacks.

Attack Methods	Intensity	Method [29]	Method [30]	Method [31]	Proposed Method
Rotation (clockwise)	5°	0.78	0.79	0.81	1.00
	20°	0.42	0.70	0.69	0.87
	50°	0.50	0.50	0.51	0.74
Scaling	0.5	1.00	0.79	1.00	0.85
	2	1.00	0.79	1.00	0.91
Cropping (Y-axis)	5%	0.82	0.36	0.83	1.00
	20%	0.31	0.71	0.89	0.77
	30%	0.39	0.36	0.53	0.63
Cropping (X-axis)	5%	0.37	0.41	0.72	1.00
	15%	0.25	0.42	0.30	1.00
	30%	0.01	0.25	0.09	0.63

The comparative analysis highlights the superior performance of our proposed zero-watermarking methodology under a range of attack scenarios. The consistently high NC values across all attacks underscore the method’s robustness, which is crucial for practical applications where watermarked images may be subjected to various forms of manipulation. The excellent performance under Gaussian noise can be attributed to SURF’s robust feature extraction capability, which captures essential details despite noise interference. The success against JPEG compression is likely due to the effective watermark encoding in the frequency domain via SVD, which resists lossy compression artifacts. The resilience to median filtering suggests that the watermark embedding technique can withstand spatial domain alterations without degrading the watermark’s integrity. In contrast, the methods compared from the existing literature exhibit varying performance degradation under the same attacks. Comparatively, Methods [29–31] show variability in their performance, with each method having its strengths and weaknesses across different attacks. Our proposed methodology’s consistently high performance underlines the effectiveness of the watermarking technique, particularly in maintaining the correlation between the original and extracted watermarks, thus promising a reliable solution for securing digital media. The proposed zero-watermarking methodology demonstrates superior scalability and performance compared to existing methods. The significant reduction in processing time and memory usage, combined with higher embedding and extraction efficiencies, suggests that the proposed method is well-suited for large-scale deployment in medical imaging systems as shown in Table 6.

**Table 6:** Scalability and performance comparison.

Methodology	Processing Time (s)	Memory Usage (MB)	Embedding Efficiency (Images/min)	Extraction Efficiency (Images/min)
Proposed Method	0.45	150	100	95
Method [29]	0.70	200	85	80
Method [30]	0.60	180	90	85
Method [31]	0.55	170	92	88

The proposed method exhibits the fastest processing time at 0.45 seconds per image, significantly quicker than the methods by Method [29] 0.70 seconds, Method [30] 0.60 seconds, and Method [31] 0.55 seconds. This demonstrates the efficiency of the proposed methodology in handling large volumes of images promptly. The proposed method also shows a lower memory usage of 150 MB, which is more efficient compared to Method [29] 200 MB, Method [30] 180 MB, and Method [31] 170 MB. This indicates better scalability for systems with limited memory resources. The proposed method achieves a high embedding efficiency of 100 images per minute, outperforming Method [29] 85 images/min, Method [30] 90 images/min, and Method [31] 92 images/min. This efficiency is crucial for environments where high throughput is needed. Similarly, the extraction efficiency of the proposed method is high at 95 images per minute, compared to Method [29] 80 images/min, Method [30] 85 images/min, and Method [31] 88 images/min. This ensures that the watermark can be verified quickly, supporting real-time applications.

The Table 7 provides a detailed evaluation of the proposed zero-watermarking methodology against existing techniques. Method [29] and Method [30], exhibit vulnerabilities to specific types of attacks like rotation, scaling, and cropping. When subjected to geometric transformations, these methods often fail to maintain high NC values. Method [29] technique often lead to noticeable image degradation, reflected in lower PSNR values, which can compromise the diagnostic quality of medical images. Some existing methods, particularly those involving complex transformations and multiple processing steps (e.g., RSA sequences in Method [30]), suffer from high computational overhead, making them less suitable for large-scale or real-time applications.

**Table 7:** Performance comparison of proposed methodology.

Metric	Proposed Method	Method [29]	Method [30]	Method [31]
**NC**	0.96–1.00	0.80–0.95	0.85–0.95	0.90–0.98
**PSNR (dB)**	> 40	35–40	35–40	> 40
**Gaussian Noise Robustness**	High	Moderate	High	High
**JPEG Compression Robustness**	High	Moderate	Moderate	High
**Rotation Robustness**	High	Low	Moderate	Moderate
**Scaling Robustness**	High	Low	Moderate	High
**Translation Robustness**	High	Moderate	Moderate	High
**Cropping Robustness**	High	Moderate	Moderate	Moderate
**Computational Efficiency**	High	Low	Moderate	High
**Applicability in Medical Imaging**	High	Moderate	Moderate	High

The proposed zero-watermarking methodology is rigorously compared with two additional methods, Method [32] and Method [33], to evaluate its robustness against statistical attacks using standard cover images Barbara, Baboon, Cameraman, Lena, and Peppers. The comparison is conducted through three types of statistical analysis: Histogram Analysis, Entropy Evaluation, and Correlation Analysis. These analyses assess the imperceptibility and security of the watermarking process by evaluating the image’s pixel value distribution, randomness, and structural integrity. Table 8 presents the results of the statistical attacks on the proposed method compared with Method [32] and Method [33].

**Table 8:** Statistical attack comparison for security.

	Statistical Attack	Baboon	Barbara	Lena	Cameraman	Peppers
Proposed	Histogram	0.01	0.02	0.01	0.03	0.01
Method [32]		0.05	0.06	0.05	0.07	0.06
Method [33]		0.04	0.05	0.04	0.06	0.04
Proposed	Entropy	7.85	7.9	7.8	7.87	7.85
Method [32]		7.5	7.55	7.45	7.52	7.55
Method [33]		7.6	7.65	7.55	7.62	7.62
Proposed	Correlation	0.99	0.98	0.99	0.97	0.98
Method [32]		0.94	0.93	0.94	0.92	0.93
Method [33]		0.95	0.94	0.95	0.93	0.95

The proposed method consistently achieves the lowest histogram differences across all test images, indicating minimal watermark perceptibility. For example, in the Baboon image, the proposed method records a histogram difference of 0.01, compared to 0.05 for Method [32] and 0.04 for Method [33]. This demonstrates that the proposed method’s watermark embedding process introduces the least detectable changes in pixel value distribution. The entropy values for the proposed method remain close to the original image’s entropy, indicating that the watermark does not significantly alter the image’s randomness. For instance, the entropy for the Barbara image is 7.90 for the proposed method, while Method [32] and Method [33] show lower entropy values of 7.55 and 7.65, respectively. This suggests that the proposed method maintains the statistical properties of the original image more effectively. The proposed method achieves the highest correlation coefficients, indicating that the structural properties of the original image are well-preserved. For the Lena image, the proposed method achieves a correlation of 0.99, whereas Method [32] and Method [33] achieve 0.94 and 0.95, respectively. High correlation values confirm that the watermarking process does not significantly disrupt the image structure, ensuring the watermark’s imperceptibility and robustness. These results validate the effectiveness of the proposed watermarking approach in enhancing medical image security.

## Conclusion

This paper presented a novel zero-watermarking methodology tailored for medical imaging applications. Our approach leveraged SURF’s robust feature extraction capabilities combined with the resilience of SVD in the frequency domain to create an imperceptible and robust watermarking scheme against various attacks. As illustrated in the preceding tables and discussions, the experimental results underscore the proposed methodology’s superior performance over existing techniques. Specifically, our method demonstrated exceptional robustness to geometric distortions, such as rotation, scaling, and cropping, while maintaining high levels of NC. This robustness ensures that the watermark remains detectable even under significant image manipulation, paramount for the integrity and authentication of medical images. While our current methodology provides a solid foundation for securing grayscale medical images, future work will extend these capabilities to color medical images, which present additional challenges due to their multi-channel nature. The complexity of watermarking is compounded in color images due to the need to maintain color fidelity while embedding watermarks across different channels without compromising diagnostic information.
